# Ventricular tachycardia ablation guided or aided by scar characterization with cardiac magnetic resonance: rationale and design of VOYAGE study

**DOI:** 10.1186/s12872-022-02581-1

**Published:** 2022-04-14

**Authors:** Alessio Lilli, Matteo Parollo, Lorenzo Mazzocchetti, Francesco De Sensi, Andrea Rossi, Pasquale Notarstefano, Amato Santoro, Giovanni Donato Aquaro, Alberto Cresti, Federica Lapira, Lorenzo Faggioni, Carlo Tessa, Luca Pauselli, Maria Grazia Bongiorni, Antonio Berruezo, Giulio Zucchelli

**Affiliations:** 1 Cardiology Division, Azienda USL Toscana Nord-Ovest, Versilia Hospital, Lido di Camaiore, Italy; 2 Second Division of Cardiology, Cardiothoracic and Vascular Department, Azienda Ospedaliero Universitaria Pisana, Via Paradisa 2, 56124 Pisa, Italy; 3 Cardiology Department, Azienda USL Toscana Sud-Est, Misericordia Hospital, Grosseto, Italy; 4 Fondazione Toscana Gabriele Monasterio, Pisa, Italy; 5 Cardiovascular Department, Azienda USL Toscana Sud-Est, San Donato Hospital, Arezzo, Italy; 6 Division of Cardiology, Azienda Ospitaliero Universitaria Senese, Siena, Italy; 7 Cardiology Division, Azienda USL Toscana Nord-Ovest, Spedali Riuniti, Livorno, Italy; 8 Department of Translational Research, University of Pisa, Pisa, Italy; 9 Division of Radiology, Azienda USL Toscana Nord-Ovest, Versilia Hospital, Lido di Camaiore, Italy; 10 Department of Radiology, Azienda USL Toscana Sud-Est, San Donato Hospital, Arezzo, Italy; 11 Arrhythmia Department, Heart Institute, Teknon Medical Center, Barcelona, Spain

**Keywords:** Ventricular arrhythmias, Ventricular tachycardia, Ventricular tachycardia ablation, Cardiac magnetic resonance, Structural heart disease, Artificial intelligence, Substrate ablation, Image-guided ablation

## Abstract

**Background:**

Radiofrequency ablation has been shown to be a safe and effective treatment for scar-related ventricular arrhythmias (VA). Recent preliminary studies have shown that real time integration of late gadolinium enhancement cardiac magnetic resonance (LGE-CMR) images with electroanatomical map (EAM) data may lead to increased procedure efficacy, efficiency, and safety.

**Methods:**

VOYAGE is a prospective, randomized, multicenter controlled open label study designed to compare in terms of efficacy, efficiency, and safety a CMR aided/guided workflow to standard EAM-guided ventricular tachycardia (VT) ablation. Patients with an ICD or with ICD implantation expected within 1 month, with scar related VT, suitable for CMR and multidetector computed tomography (MDCT) will be randomized to a CMR-guided or CMR-aided approach, whereas subjects unsuitable for imaging or with image quality deemed not sufficient for postprocessing will be allocated to standard of care ablation. Primary endpoint is defined as VT recurrences (sustained or requiring appropriate ICD intervention) during 12 months follow-up, excluding the first month of blanking period. Secondary endpoints will include procedural efficiency, safety, impact on quality of life and comparison between CMR-guided and CMR-aided approaches. Patients will be evaluated at 1, 6 and 12 months.

**Discussion:**

The clinical impact of real time CMR-guided/aided ablation approaches has not been thoroughly assessed yet. This study aims at defining whether such workflow results in more effective, efficient, and safer procedures. If proven to be of benefit, results from this study could be applied in large scale interventional practice.

*Trial registration*ClinicalTrials.gov, NCT04694079, registered on January 1, 2021.

## Background

Malignant ventricular arrhythmias (VA) represent “electrical abnormalities” of the heart that are the final result of several myocardial diseases and the leading cause of sudden death in cardiac patients [1].

Previous studies and meta-analyses [2, 3] clearly demonstrated that the presence of a myocardial scar is a significant predictor of sudden death, malignant arrhythmias or ICD shocks. The presence of isles and channels of viable myocardium represents the main pathological basis of most arrhythmia-related scars. Several triggers such as heart failure, molecular changes or autonomic nervous system unbalances have been considered important in the clinical development of arrhythmias [4]. Nevertheless, the substrate linked to jeopardized scar and viable myocardium constitutes the root of reentry circuits and, importantly, a major target for therapy. Every single subject will develop, after a myocardial injury, a specific pattern of fibrosis, with some individual, unpredictable characteristics (endo/epicardial involvement, confluent, jeopardized or merged fibrosis with normal tissue).

In the last decade, effort has been made to change the perspective of approach to VA and sudden cardiac death. ICD became the cornerstone of management; however, no event can be prevented by ICD, that effectively treats VAs only after their onset. Pharmacological therapy comprises only few drugs that interfere directly with the electrical properties of the myocardium usually with a single mechanism for every molecule. Unfortunately, major adverse effects of drugs limit their use and mitigate their benefit [5]. Consequently, percutaneous ablation becomes an important approach to prevent VA occurrence [6, 7].

The traditional approach to VT ablation implies the recognition of the origin and/or propagation of the myocardial signal during arrhythmia. Mapping of myocardial potentials during spontaneous rhythm or induced arrhythmias helps the physician to recognize possible foci or circuits for propagation. In a recent meta-analysis, the combined risk of ventricular arrhythmia recurrence and all-cause mortality during long-term follow-up was lower when using a substrate-based approach compared to standard ablation of stable VT. However, efficacy was greatly reduced if the substrate modification was incomplete [8].

Given the highly patient-specific pattern of myocardial fibrosis and the importance of a substrate-oriented approach, we highlight the opportunity to individualize the diagnostic and therapeutic strategies.

This goal can be attained by means of substrate identification, anatomic reconstruction (i.e., mapping of the fibrosis) and tailored modification. From a clinical point of view, cardiac magnetic resonance for the substrate characterization and radiofrequency ablation represent the mainstream “tools” to achieve the purpose.

The aim of the VOYAGE (Ventricular tachycardia ablatiOn and mYocardial scAr chracterization with maGnetic rEsonance) trial (ClinicalTrials.gov NCT04694079) is to test, in terms of efficacy, safety and efficiency, a tailored approach to VT ablation. The study will compare current standard of care with a tailored approach for VA radiofrequency (RF) ablation. The experimental approach is based on pre-interventional anatomic substrate definition by Cardiac Magnetic Resonance (CMR) with Late Gadolinium Enhancement (LGE), using a post-processing 3D elaboration software.

## Methods

### Study hypothesis and primary endpoint

Our hypothesis is that a comprehensive preprocedural individual characterization of the myocardial substrate via a CMR scan, and its integration within the mapping system can significantly improve technical intervention and patient outcome. The primary objective is to analyze the outcome of CMR guided/aided approaches of VT ablation in terms of effectiveness at 12 months in comparison to a control group (standard of care VT ablation). The primary endpoint is defined as any VT recurrences during a 12-months follow-up (not including the first month). VT episodes will be considered as recurrences if sustained (more than 30 s) or requiring appropriate ICD intervention and will be detected during outpatient clinic evaluation through an ICD interrogation.

Pre-specified group subanalyses will compare in terms of the primary endpoint:

- Patients with structural heart disease of ischemic origin
- Patients undergoing endocardial-substrate only ablation

Secondary objectives are shown in Table 1.

**Table 1:** Secondary endpoints

Secondary endpoints
Health-related quality of life changes
Procedural time (introducer in-introducer out)
Fluoroscopy duration
X-ray exposure
Radiofrequency time
Number of RF applications
VT inducibility at the end of ablation with number of residual VTs
Appropriate and inappropriate ICD interventions (ATP or shocks) anytime and after 1 month
Rate of patients with contraindication to CRM or with poor CMR imaging
Hospital admission for cardiac causes
VT storm anytime and after 1 month
Composite endpoint including death at any time, VT storm and appropriate ICD shock after 1 month of treatment
*Complications during 12-month follow-up*
Death (related or not to the procedure)
Myocardial infarction
Stroke or transient ischemic attack (TIA)
Other thromboembolic events
Cardiac perforation or tamponade
Vascular complications
Prolonged or repeat hospitalization
Heart block
Pericarditis requiring intervention
Pneumothorax
Pulmonary edema
Hepatic injury
Abdominal issues

### Trial design

The VOYAGE trial is a prospective, randomized, multicenter open-label study with control group involving a total of eight centers. The research consortium includes both Academic (2 centers: Azienda Ospedaliero Universitaria Pisana e Azienda Ospedaliero Universitaria Senese) and non-Academic (6 centers: 2 located at Azienda Sanitaria Locale Toscana Nord Ovest, 2 at Azienda Sanitaria Locale Sud Est, 1 at Fondazione Toscana G. Monasterio, 1 at Teknon Medical Center—Heart Institute) hospitals located in Italy (n = 7) and Spain (n = 1). Some centers are involved in patient recruitment and cardiac imaging evaluation whereas others are also equipped for ablation procedures (5 centers). Teknon Medical Center – Heart Institute (Barcelona, Spain) is involved as an external research organization, with a training and technical support role, in particular regarding imaging dataset acquisition, post-elaboration and integration during ablation procedures. Centre will also participate on data analysis and result dissemination. The coordinating center is an Academic center located in Pisa (Azienda Ospedaliera Universitaria Pisana) with expertise in ventricular ablation procedures.

The study provides three arms:

- Group 1, Experimental: CMR guided ablation of the "anatomical" channels based on anatomical scar
- Group 2, Experimental: CMR aided ablation of the "electrical" conducting channels (CCs) within the scar
- Group 3, Standard of Care: Ablation guided by an electro-anatomical system

**Fig. 1:**
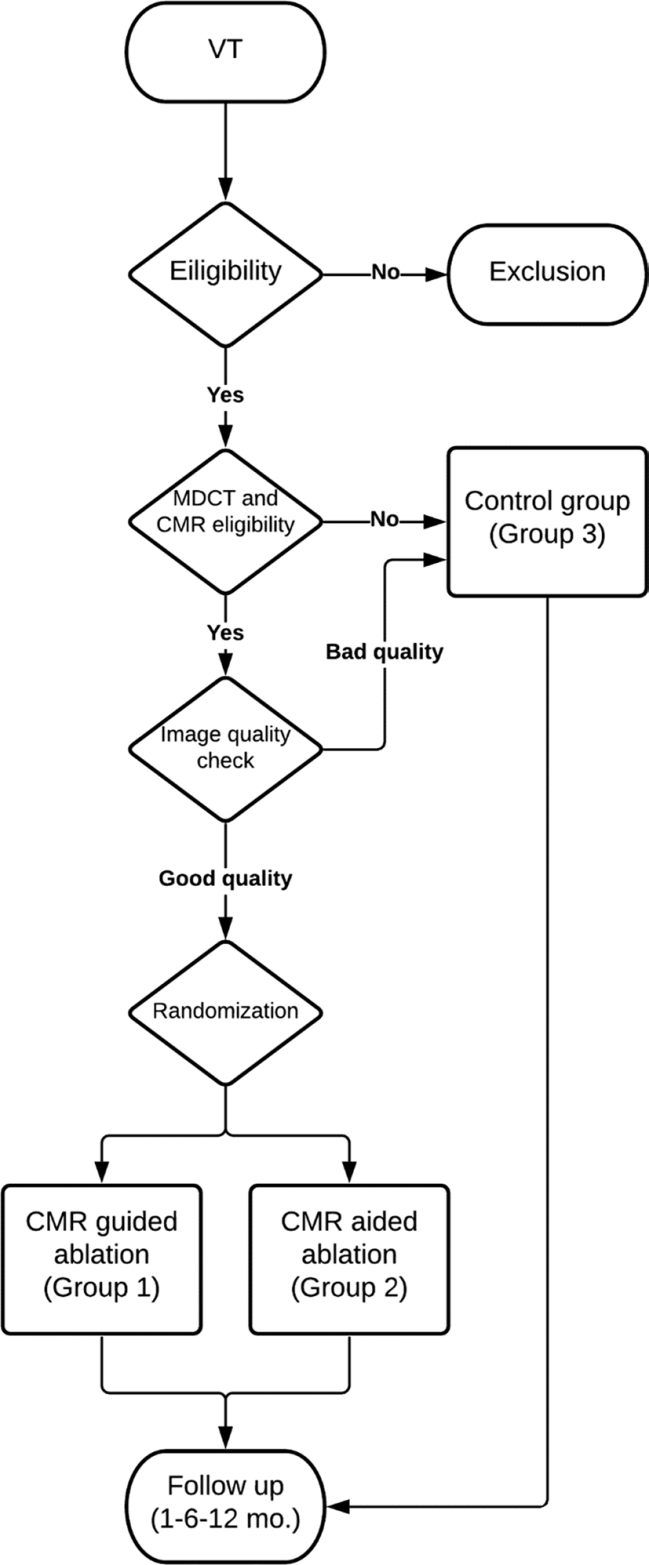
Study design flowchart. VT: ventricular tachycardia; MDCT: multidetector computed tomography; CMR: cardiac magnetic resonance

All patients who give informed consent for participation and fulfill the inclusion eligibility criteria will be consecutively enrolled (Inclusion and exclusion criteria are respectively shown in Tables 2 and 3). They will subsequently be evaluated for eligibility to CMR and CT. Patients with clinical or device-related contraindications to CMR or CT or with image quality deemed suboptimal for postprocessing will be assigned to Group 3 and undergo standard of care ablation. The other patients will be randomized in a 1:1 fashion to either Group 1 or 2 using a web-based platform. The scientific coordinator of the study or a delegate should be the unique responsible of randomization and will be contacted upon all new recruitments. LGE-CMR data obtained by 1.5 or 3 T CMR and multi-detector cardiac tomography (MDCT) data obtained using a 64 to 128 slice CT scanner will be processed with a specific commercial software (ADAS 3D, Galgo Medical, Barcelona, Spain). In Group 1, ablation will be performed based only on cardiac imaging information processed by the abovementioned software without electroanatomical mapping. A so-called anatomical approach is consistent with a previous observation [9] that ablation of channels only can be effective in preventing VA recurrences. In Group 2 ablation procedure will be based on information derived from both imaging and electroanatomic mapping data, but electrical CCs will remain as the final ablation target. Finally, Group 3 will be treated with standard ablation according to current clinical practice based on electroanatomic mapping (EAM). (Fig. 1).

**Table 2:** Study inclusion criteria

Inclusion criteria
Indication for VT ablation in patients with structural heart disease (indications according to the 2015 ESC Guidelines for the management of patients with ventricular arrhythmias and the prevention of sudden cardiac death);
Structural heart disease (clinical history, ECG, multimodality imaging) involving the left ventricle
Signed informed consent;

**Table 3:** Study exclusion criteria

Exclusion criteria
Age < 18 years;
ICD not already implanted nor expected within 1 month;
High probability of non-adherence to the follow-up requirements (due to social, psychological, or medical reasons);
Inability to give written informed consent;
Pregnancy (suspected or confirmed);
Acute coronary syndrome or PCI in the previous 30 days
Creatinine clearance < 15 ml/min (stage 5 CKD) (according to clinical history or out/in-patient tests performed upon enrollment)
Severe chronic liver disease (Child–Pugh score C) (according to clinical history or out/in-patient tests performed upon enrollment)
Heart surgery for valve disease in the previous 6 months or expected within 6 months
Coronary artery bypass graft in the previous 3 months
NYHA functional class IV heart failure or CCS functional class IV angina
Previous VT ablation
Systemic illness likely to limit survival to < 1 year

### Preprocedural CT scan

In absence of contraindications, a contrast-enhanced multi-detector cardiac tomography (MDCT) will be obtained using a 64 or 128 slice CT scanner, with ECG gating set between 50 and 100% of cardiac cycle. The exam will be performed after hospital admission or in an outpatient setting. Anatomical data obtained from such exam will be used for clinical management purposes and, after post-elaboration with ADAS 3D software, integrated within the spatial reference coordinates of the EAM system (CARTO 3 Biosense Webster, Diamond Bar, CA, USA), alongside data derived from LGE-CMR.

### Preprocedural CMR scan

In absence of contraindications, an LGE-CMR will be obtained using a 1.5 or 3 T scanner. In patients already implanted with an MRI-conditional ICD, the exam will be performed using a 1.5 T scanner using a specific wideband sequence in order to avoid device-related artifacts. CMR machines in use at participating centres at time of publication are: Magnetom Avanto 1.5 T (Siemens Healthcare, Erlangen, Germany), Magnetom Aera 1.5 T (Siemens Healthcare, Erlangen, Germany) and Signa Artist 1.5 T (GE Healthcare, Chicago, IL, USA). Anatomical data obtained from such exams will be used for clinical management purposes and, after post-elaboration with ADAS 3D software, integrated within the spatial reference coordinates of the electroanatomical mapping system (CARTO 3 Biosense Webster, Diamond Bar, CA, USA), alongside anatomical data derived from MDCT.

### Image processing

Images processing (ADAS 3D Galgo Medical, Barcelona, Spain) is pivotal to translate radiomics data produced by CMR into utilizable maps within the electrophysiology lab. Briefly, sequence packs created within each center will be transferred and processed by core-lab and finally elaborated by the software with the purpose of being integrated with the ablation mapping suite (CARTO 3, Biosense Webster, Diamond Bar, CA, USA). A pre-study phase for the achievement of quality standards in images acquisition has been accomplished in every center before the enrollment start date. The core-lab for image processing (U.O. Cardiologia 2—Azienda Ospedaliero Universitaria Pisana), will be further supported, if required, by Heart Institute in Teknon Medical Center (Barcelona, Spain). A VPN tunnel and an SFTP server will be used to securely transfer anonymized data between centers for post-elaboration.

LV endocardial and epicardial borders will be delimited by an experienced operator with a semiautomatic segmentation algorithm. Nine concentric layers will be identified from endocardium to the epicardium (10 to 90% of wall thickness). LGE-CMR information will be projected using trilinear interpolation over each layer surface and signal intensity distribution will be color-coded. Scar core (SC) and border zones (BZ) will be defined as 40% ± 5% and 60% ± 5% of maximum signal intensity respectively. Heterogeneous tissue channels (HTCs) will be defined as a corridor of BZ between two core areas or between a core area and a valvular annulus connecting two areas of healthy tissue.

The software will identify HTCs automatically, and they will be classified as:

1. Sub-endocardial (layers from 10 to 50%).
2. Sub-epicardial (layers from 60 to 90%).
3. Transmural (layers from 10 to 90%).

Pixel signal intensity (PSI) maps and HTCs derived from LGE-CMR studies will subsequently be fused and aligned with the anatomical reconstructions extracted from multidetector cardiac tomography (MDCT) using the ADAS 3D software platform. This alignment allows the integration within the spatial reference coordinates of the mapping system by performing a fast anatomical mapping of the aortic root [9].

### Ablation procedure

Ablation procedure will be carried out in an electrophysiology lab by using an electroanatomical mapping system (CARTO 3, Biosense Webster, Diamond Bar, CA, USA). An open irrigated 3.5 mm tip radiofrequency catheter (ThermoCool SmartTouch SF, Biosense Webster, Diamond Bar, CA, USA) will be used both for mapping and ablation.

In Groups 1 and 2 fast anatomical mapping (FAM) of the aorta from the aortic root to the origin of the left subclavian artery will be obtained in order to align such reconstruction with the MDCT-derived 3D model of the aorta and left ventricle, thus enabling the alignment between CARTO spatial references and post-processed CMR derived 3D model.

An electroanatomical map (EAM) of the target chamber will be obtained only in groups 2 and 3. EGMs will however be analyzed in all cases to avoid applications:

1. At the level of His or fascicular EGMs
2. At the level of EGMs with an amplitude > 3 mV or 1.5–3 mV without delayed components, during sinus rhythm of after application of multiple extrastimuli to check for the presence of hidden slow conduction.

The target for RF ablation changes accordingly to the group of allocation or randomization.

**Fig. 2:**
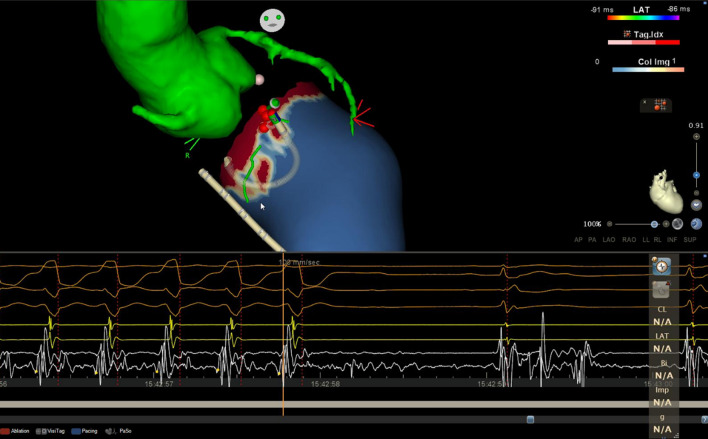
Mapping suite showing a CMR-guided procedure, with VT interruption during RF delivery at an heterogeneous tissue channel **(**HTC**)** on 3D CMR model. Green: MDCT-derived aorta 3D model, blue areas: healthy tissue, red areas: scar tissue, yellow areas: border zone

Group 1: RF energy will be delivered at the entrance of HTCs identified in the maps created by images processing, regardless of the presence of any pathological electrogram (EGM) at such locations (Fig. 2). If VT is spontaneously induced, it’s allowed to map the circuit and ablate during tachycardia, even though it’s suggested to acquire the template and stop the arrhythmia (at first appearance) and continue the ablation of substrate.

Group 2 and Group 3: Ablation sites will be identified after analyzing the complete EAM, targeting conducting channel entrance-compatible EGMs, according to the “scar dechanneling” technique [10]. Other techniques aimed at achieving complete substrate ablation and currently used by the centre performing VT ablation, will be accepted. In case of absence of electrograms with delayed components at EAM, VT will be induced, and mapping will be done to delineate the circuit. In Group 2 the scar area localization via CMR images integrates the EAM data to help in substrate delineation focusing on that area. In Group 3 a complete left ventricle EAM will be achieved.

At the end of ablation, we will verify the absence of pathological EGMs and we’ll proceed towards inducibility test. In case of VT induction, we could have 2 scenarios: (1) hemodynamically tolerated VT: it will be mapped and ablated; (2) not hemodynamically tolerated VT: VT template will be taken and, after its interruption, used for pacemapping to identify VT exit site, which will undergo ablation.

Acute success will be defined as non-inducibility (2 drives, 2 sites, up to 3 extrastimuli) of any sustained monomorphic VT at the end of the procedure. Partial success will be considered with the clinical VT successfully ablated with other monomorphic VTs remaining inducible. Pre-ablation inducibility is not required and is discouraged unless in the abovementioned conditions.

### Device programming and medical therapy

All patients involved in the study, regardless of Group allocation, will have their ICD programmed following 2019 HRS/EHRA/APHRS/LAHRS expert consensus statement on optimal ICD programming [11], following the manufacturer specific recommendations about therapy and detection in patients where VT cycle is known, alongside any anti-bradycardia setting necessary for therapeutic needs. Particularly, the VT zone with lower rate detection should be set at 10–20 beats/min slower than the clinical VT to guarantee a good sensitivity in recurrences diagnosis and therapy should be programmed in order to reduce unnecessary shocks.

Patients involved in the study will be treated with antiarrhythmic drugs and any other medical therapy considered standard of care for the SHD and VT. Particularly, use of amiodarone is allowed at enrollment but it is strongly suggested to stop it at 1 month follow-up, if not contraindicated, to avoid drug-related toxicity.

### Follow-up

Clinical follow-up (FU) will include outpatient clinic visits at 1, 6 and 12 months after the ablation procedure. If no contraindications, amiodarone (if used) should be stopped at the 1-month FU visit. At all follow up visits, the following parameters and information will be taken:

- Physical examination.
- 12-lead surface ECG.
- Transthoracic echocardiography (at 6 and 12 months).
- ICD interrogation, analyzing any event marked as VT or VF by the device.

To assess changes in health-related quality of life, the 36-Item Short Form Survey (SF-36) will be administered at enrolment and at the 12-months follow up visit (Table 4).

**Table 4:** Study timeline

PROCEDURE	VISIT						
	Enrollment	Baseline	Ablation	Discharge	1 month	6 months	12 months
Eligibility screen	✓						
Informed consent	✓						
Demographic Data		✓					
Medical History		✓					
QoL assessment		✓					✓
Medication		✓		✓	✓	✓	✓
12-lead ECG		✓		✓	✓	✓	✓
CMR (gr. 1 and 2)		✓					
TTE		✓				✓	✓
Cardiac CT (gr. 1 and 2)		✓					
ICD check				✓	✓	✓	✓
Ablation data			✓				
Safety data			✓	✓	✓	✓	✓
Protocol Deviation	✓	✓	✓	✓	✓	✓	✓
Adverse event	✓	✓	✓	✓	✓	✓	✓
Termination	✓	✓	✓	✓	✓	✓	✓

### Sample size and statistical analysis

To test the primary hypothesis, 103 patients will be necessary with 80% power and 5% type 1 error, considering 50% of group 1–2 (low risk), 50% of group 3 (high risk), 16% of likelihood of VT recurrences at 12 months in low-risk patients and 44% of likelihood in high-risk patients with HR (high risk/low risk of recurrences) of 2.75 (Cox PH, 2-sided equality).

All applicable statistical tests will be 2-sided and will be performed using a 5% significance level. Continuous variables will be reported as mean ± standard deviation, or median (range or interquartile range if data are skewed) if not normally distributed; these variables will be compared using Student’s t-test if normally distributed, Aspin-Welch test if normally distributed with unequal variance demonstrated using Variance-ratio F-test, or Mann–Whitney U test if not normally distributed. Categorical variables will be expressed as total number (percentage) and will be compared by chi-squared test. As far as the primary endpoint, data will be analyzed according to the intention-to-treat principle. Survival free from ventricular arrhythmia, will be evaluated using a time-to-first-event analysis with the Logrank test and the Kaplan–Meier cumulative event rate. A multivariable Cox proportional hazards model will be performed to investigate the effects of baseline characteristics in predicting ablation results.

## Discussion

Integration of high-definition myocardial substrate data acquired via imaging techniques such as CMR and MDCT has already been proved as a feasible and potentially effective solution for VT ablation, even in complex and cutting-edge scenarios [9, 12–14]. However, MDCT-only approaches, in which myocardial wall thickness maps can identify "CT-channels" [15] where VT functional isthmuses can be located, showed poor performance [16] when compared to CMR in correctly identifying the arrhythmogenic substrate, especially when entirely subendocardial.

The mainstay of our proposal is represented by real time integration, alignment, and synchronization of 3D-meshes, containing anatomical data obtained from MDCT, and substrate data derived from postprocessed CMR, with the tridimensional references of the electroanatomical mapping suite, allowing operators to effectively navigate such meshes irrespectively of the presence of a standard complete LV EAM.

As far as CMR is concerned, single-center experiences have already shown that an approach where MRI derived information is used alongside EAM data, to aid VT ablation ("MRI-aided"), may lead to improved efficacy over traditional EAM-only techniques [12, 17]. The natural evolution of such method may be represented by “MRI-guided” approaches, where EAM acquisition is completely avoided, and the operator relies solely on imaging data; a single-center nonrandomized study demonstrated that this approach is not only feasible and safe, but could lead to lower acute VT inducibility rates and comparable long-term efficacy with “MRI aided” procedures, while allowing more efficient procedures, with lower procedure duration and radiological exposure [9].

The primary objective of this study is to verify, for the first time in a multicenter controlled open-label clinical trial, whether CMR guided and aided strategies, as a whole, truly represent a more effective, more efficient, and at least as safe, if not safer, VT ablation approach when compared to the standard EAM-only guided technique. A confirmation of our primary hypothesis can also lead to the speculation that an imaging-based workflow could better standardize the approach to VT’s substrate, finally determining less variability between operators and a smoother) VT ablation learning curve, as compared to EAM only technique.

While not powered for this specific objective, our study could also reveal whether a complete anatomical CMR-derived substrate elimination, as established for CMR-guided (“Group 1”) procedures, could guarantee better long-term results. The present trial was designed to overcome most of limitations related to the implementation of our proposed approach . First, the incidence of treatable VA is relatively low in general population and high volume (of cases) is critical for both imaging elaboration and ablation procedures. Accordingly, we created a network of centers, each with at least one major expertise, and a period of training before the trial (6 months) has been devoted to share information about image acquisition and ablation approaches. Eventually, a total of four centers for CMR acquisition and five centers for ablation were able to provide high quality activities, with a definite standardization of CMR sequences and treatment. The most critical process, represented by standardization of imaging acquisition protocols, has been guaranteed and supervised by the core laboratory, in collaboration with the aforementioned external research organization.

In conclusion, VOYAGE is the largest trial so far to propose and test, in a randomized setting, a comprehensive workflow for CMR-guided/aided VT ablation. If positive, results from this study could be applied in large scale in the electrophysiology practice, contributing to the adoption of substrate-tailored approaches, coherently with current trends in the era of precision medicine.

## Trial status

Patient recruitment started in June 2021 and is ongoing at time of publication. Enrollment completion is estimated by June 2023. The 12-month follow-up information of the last included patient should be available approximately 3 years after the first inclusion.

## Data Availability

Not applicable. Enrollment is currently ongoing, and no datasets were generated for analysis yet.

## Abbreviations

BZ: Border zone
CCS: Canada Cardiovascular Society
CKD: Chronic kidney disease
CMR: Cardiac magnetic resonance
CT: Cardiac tomography
EAM: Electroanatomical map
ECG: Electrocardiogram
FAM: Fast anatomical mapping
FU: Follow up
HR: Hazard ratio
HTC: Heterogeneous tissue channel
ICD: Implantable cardioverter-defibrillator
LGE: Late gadolinium enhancement
MDCT: Multi-detector cardiac tomography
NYHA: New York Heart Association
PCI: Percutaneous coronary intervention
PSI: Pixel signal intensity
RF: Radiofrequency
SC: Scar core
SF-36: 36-Item Short Form Survey
SFTP: SSH file transfer protocol
SHD: Structural heart disease
TTE: Transthoracic echocardiography
VA: Ventricular arrhythmia
VF: Ventricular fibrillation
VPN: Virtual private network
VT: Ventricular tachycardia

## References

[1] Priori SG, Blomström-Lundqvist C, Mazzanti A, Blom N, Borggrefe M, Camm J, Elliott PM, Fitzsimons D, Hatala R, Hindricks G, Kirchhof P, Kjeldsen K, Kuck K-H, Hernandez-Madrid A, Nikolaou N (2015). 2015 ESC Guidelines for the management of patients with ventricular arrhythmias and the prevention of sudden cardiac death: the task force for the management of patients with ventricular arrhythmias and the prevention of sudden cardiac death of the European Society of Cardiology (ESC) endorsed by: Association for European Paediatric and Congenital Cardiology (AEPC). Eur Heart J.

[2] Disertori M, Rigoni M, Pace N, Casolo G, Masè M, Gonzini L, Lucci D, Nollo G, Ravelli F (2016). Myocardial fibrosis assessment by LGE is a powerful predictor of ventricular tachyarrhythmias in ischemic and nonischemic LV dysfunction: a meta-analysis. JACC Cardiovasc Imaging.

[3] Sánchez-Somonte P, Quinto L, Garre P, Zaraket F, Alarcón F, Borràs R, Caixal G, Vázquez S, Prat S, Ortiz-Perez JT, Perea RJ, Guasch E, Tolosana JM, Berruezo A, Arbelo E (2021). Scar channels in cardiac magnetic resonance to predict appropriate therapies in primary prevention. Heart Rhythm.

[4] Shivkumar K (2019). Catheter ablation of ventricular arrhythmias. N Engl J Med.

[5] Bokhari F, Newman D, Greene M, Korley V, Mangat I, Dorian P (2004). Long-term comparison of the implantable cardioverter defibrillator versus amiodarone: eleven-year follow-up of a subset of patients in the Canadian Implantable Defibrillator Study (CIDS). Circulation.

[6] Reddy VY, Reynolds MR, Neuzil P, Richardson AW, Taborsky M, Jongnarangsin K, Kralovec S, Sediva L, Ruskin JN, Josephson ME (2007). Prophylactic catheter ablation for the prevention of defibrillator therapy. N Engl J Med.

[7] Sapp JL, Wells GA, Parkash R, Stevenson WG, Blier L, Sarrazin J-F, Thibault B, Rivard L, Gula L, Leong-Sit P, Essebag V, Nery PB, Tung SK, Raymond J-M, Sterns LD (2016). Ventricular tachycardia ablation versus escalation of antiarrhythmic drugs. N Engl J Med.

[8] Briceño DF, Romero J, Villablanca PA, Londoño A, Diaz JC, Maraj I, Batul SA, Madan N, Patel J, Jagannath A, Mohanty S, Mohanty P, Gianni C, Della Rocca D, Sabri A (2018). Long-term outcomes of different ablation strategies for ventricular tachycardia in patients with structural heart disease: systematic review and meta-analysis. EP Europace.

[9] Soto-Iglesias D, Penela D, Jáuregui B, Acosta J, Fernández-Armenta J, Linhart M, Zucchelli G, Syrovnev V, Zaraket F, Terés C, Perea RJ, Prat-González S, Doltra A, Ortiz-Pérez JT, Bosch X (2020). Cardiac magnetic resonance-guided ventricular tachycardia substrate ablation. JACC Clin Electrophysiol.

[10] Berruezo A, Fernández-Armenta J, Andreu D, Penela D, Herczku C, Evertz R, Cipolletta L, Acosta J, Borràs R, Arbelo E, Tolosana JM, Brugada J, Mont L (2015). Scar dechanneling: new method for scar-related left ventricular tachycardia substrate ablation. Circ Arrhythm Electrophysiol.

[11] Stiles MK, Fauchier L, Morillo CA, Wilkoff BL (2019). 2019 HRS/EHRA/APHRS/LAHRS focused update to 2015 expert consensus statement on optimal implantable cardioverter-defibrillator programming and testing. Europace: Eur Pacing Arrhythm Cardiac Electrophysiol: J Work Groups Cardiac Pacing Arrhythmias Cardiac Cell Electrophysiol Eur Soc Cardiol.

[12] Andreu D, Penela D, Acosta J, Fernández-Armenta J, Perea RJ, Soto-Iglesias D, de Caralt TM, Ortiz-Perez JT, Prat-González S, Borràs R, Guasch E, Tolosana JM, Mont L, Berruezo A (2017). Cardiac magnetic resonance-aided scar dechanneling: Influence on acute and long-term outcomes. Heart Rhythm.

[13] Berruezo A, Penela D, Jáuregui B, Soto-Iglesias D (2021). The role of imaging in catheter ablation of ventricular arrhythmias. Pacing Clin Electrophysiol: PACE.

[14] Zucchelli G, Parollo M, Di Cori A, Matteucci F, Berruezo A, Bongiorni MG (2021). Stereotactic ventricular tachycardia radioablation aided by CT-channels analysis in a patient with inaccessible transmural substrate. EP Europace.

[15] Takigawa M, Duchateau J, Sacher F, Martin R, Vlachos K, Kitamura T, Sermesant M, Cedilnik N, Cheniti G, Frontera A, Thompson N, Martin C, Massoullie G, Bourier F, Lam A (2019). Are wall thickness channels defined by computed tomography predictive of isthmuses of postinfarction ventricular tachycardia?. Heart Rhythm.

[16] Jáuregui B, Soto-Iglesias D, Zucchelli G, Penela D, Ordóñez A, Terés C, Chauca A, Acosta J, Fernández-Armenta J, Linhart M, Perea RJ, Prat-González S, Bosch X, Ortiz-Pérez JT, Mont L (2020). Arrhythmogenic substrate detection in chronic ischaemic patients undergoing ventricular tachycardia ablation using multidetector cardiac computed tomography: compared evaluation with cardiac magnetic resonance. EP Europace.

[17] Zghaib T, Ipek EG, Hansford R, Ashikaga H, Berger RD, Marine JE, Spragg DD, Tandri H, Zimmerman SL, Halperin H, Brancato S, Calkins H, Henrikson C, Nazarian S (2018). Standard ablation versus magnetic resonance imaging–guided ablation in the treatment of ventricular tachycardia. Circ Arrhythm Electrophysiol.

